# Hypo-Osmotic Stress and Pore-Forming Toxins Adjust the Lipid Order in Sheep Red Blood Cell Membranes

**DOI:** 10.3390/membranes13070620

**Published:** 2023-06-25

**Authors:** Rose Whiting, Sevio Stanton, Maryna Kucheriava, Aviana R. Smith, Matt Pitts, Daniel Robertson, Jacob Kammer, Zhiyu Li, Daniel Fologea

**Affiliations:** 1 Department of Physics, Boise State University, Boise, ID 83725, USA; 2 Biomolecular Sciences Graduate Program, Boise State University, Boise, ID 83725, USA; 3 Department of Family Medicine, Idaho College of Osteopathic Medicine, Meridian, ID 83642, USA

**Keywords:** lipid order, hypo-osmotic shock, pore-forming toxins, Laurdan, diphenylhexatriene, phase transition

## Abstract

Lipid ordering in cell membranes has been increasingly recognized as an important factor in establishing and regulating a large variety of biological functions. Multiple investigations into lipid organization focused on assessing ordering from temperature-induced phase transitions, which are often well outside the physiological range. However, particular stresses elicited by environmental factors, such as hypo-osmotic stress or protein insertion into membranes, with respect to changes in lipid status and ordering at constant temperature are insufficiently described. To fill these gaps in our knowledge, we exploited the well-established ability of environmentally sensitive membrane probes to detect intramembrane changes at the molecular level. Our steady state fluorescence spectroscopy experiments focused on assessing changes in optical responses of Laurdan and diphenylhexatriene upon exposure of red blood cells to hypo-osmotic stress and pore-forming toxins at room temperature. We verified our utilized experimental systems by a direct comparison of the results with prior reports on artificial membranes and cholesterol-depleted membranes undergoing temperature changes. The significant changes observed in the lipid order after exposure to hypo-osmotic stress or pore-forming toxins resembled phase transitions of lipids in membranes, which we explained by considering the short-range interactions between membrane components and the hydrophobic mismatch between membrane thickness and inserted proteins. Our results suggest that measurements of optical responses from the membrane probes constitute an appropriate method for assessing the status of lipids and phase transitions in target membranes exposed to mechanical stresses or upon the insertion of transmembrane proteins.

## 1. Introduction

The cell membrane evolved from a selective barrier separating the cell from the external environment to a more complex functional structure with regulatory roles in transport, communication, and signal transduction. The ingenious arrangement of lipids proposed by the long-revered mosaic fluid model more than five decades ago is still the most fundamental model for the structure and dynamics of the plasma membrane [1]. The concept of basic lipid state phases postulated by the original model (i.e., liquid crystalline and bilayer) [2] has been refined [1,3,4,5,6] to better capture the importance of lipid organization in domains [7,8,9,10,11,12] for describing the membrane’s dynamics, regulation, and permeation. Currently, the membrane is viewed as a complex system consisting of liquid-ordered and liquid-disordered phases [5,13]. The lipid order in the membranes has physiological relevance for a large variety of fundamental biological processes: apoptosis [14], signaling [15,16,17,18], cellular development [19], interactions [20], receptor stabilization [21], the segregation and mobility of membrane proteins [22], microvesicle release [23], and clonal expansion [24]. Consequently, the assessment of changes in lipid ordering is under intense scrutiny to identify the origin of triggering factors and the cascades of biochemical and biophysical signals leading to the observed biological effects. Investigations into the organization of lipids in membranes may be experimentally approached by employing numerous biophysical techniques. Despite the variety of methodologies, the large availability of steady-state fluorescence spectroscopy instrumentation and the ease of experimental procedures led to a wide adaptation of this technique for investigating the status of membranes in natural and artificial systems [25,26,27,28,29,30,31,32].

Multiple fluorescent probes for evaluating membrane status and lipid organization are commonly used, and many alternatives have been proposed for adoption. Among these, 6-dodecanoyl-2-dimethylaminonaphtalene (Laurdan) and 1,6-diphenyl-1,3,5-hexatriene (DPH) have been extensively used as reporters in numerous natural and artificial membrane systems [26,27,33,34,35,36,37,38]. While neither probe directly responds to lipid ordering, their optical responses adjust to changes in the physical environment. Laurdan’s dipolar relaxation leads to spectral shifts [5,30,31,32,34,38,39], and DPH’s anisotropy is affected by the rotational diffusion of the dye [29,36,37]. Both mechanisms are influenced by the ordering of the lipids in the membranes in which the probes reside.

Changes in membrane status are ascribed to adjustments in numerous physical and biomechanical parameters, which are closely associated with lipid ordering [25,40,41,42,43,44,45,46]. Absolute lipid ordering is not easy to quantify. As a result, many investigations focused on assessing its variation in response to physical and chemical adjustments. The most common environmental stressor is temperature as changes in ordering are closely related to temperature-induced phase transitions. Temperature-induced phase transitions are easier to observe in artificial systems with limited components [27,28,47] and intervals that include the melting temperature of the lipids under investigation [26,27,28,47,48]. The indicative hallmark of transitions from ordered to disordered states is the sigmoidal shape of the melting curve [26,27,28]. Even in artificial systems, the addition of cholesterol dramatically changes the transition profile by adjusting the melting temperature and flattening the melting curve. Consequently, sigmoidal shapes may be observed only by pushing the temperatures well beyond physiological ranges [27,28,47]. The situation is more complicated for cell membranes, in which the composition is very complex [49,50] and may include stabilizing small molecules. Natural membranes are predominantly in a liquid-ordered phase and unable to attain an ordered gel phase within physiological temperature ranges [2]. Cholesterol additions adjust the melting temperatures and smooth the transitions as a result of lipid ordering elicited by cholesterol [26,28,51,52]. However, this makes data interpretation difficult. One example comes from attempts to decipher the effects of osmotic pressure on membrane properties. In this respect, several reports indicate specific changes in lipid ordering, but these investigations employed either artificial membranes or hyperosmotic shock [35,53,54,55]. These experiments demonstrated that hyperosmotic conditions lead to increased rigidity, ordering, and transition temperature, while hypo-osmotic shock experiments that employed *S. cerevisiae* show membrane fluidization [56]. In addition to assessing the effects of environmental factors, a different direction of research focuses on understanding the effects on ordering of various inclusions into membranes, which is highly relevant for protein insertion, assembly, and biological functionalities.

Despite numerous advancements in establishing relationships between the influence of physical, chemical, and biological factors on lipid organization and biological consequences, little is known of the influence of hypo-osmotic pressure or the insertion of transmembrane proteins on ordering in natural membranes. To fill these gaps in our knowledge, we employed fluorescence techniques to measure optical responses of Laurdan and DPH on red blood cell (RBC) membranes exposed to hypo-osmotic shocks and increasing concentrations of pore-forming toxins (PFTs).

We concluded that hypo-osmotic shocks and PFTs acting at room temperature gradually decrease the lipid ordering to an extent comparable with what is customarily observed for phase transitions. The effects of hypo-osmotic shocks are interpreted by considering the stretching of the membranes, while the effects of protein insertions are explained based on local changes in geometry and bending resulting from hydrophobic mismatches.

## 2. Materials and Methods

### 2.1. Red Blood Cell Preparation

Freshly isolated sheep RBCs in Alsever’s solution (Colorado Serum Company, Denver, CO, USA) were utilized in our experiments. The RBCs were purified by repeated centrifugation (3500 RPM, five minutes, Fisher Scientific bench centrifuge, ThermoFisher Scientific, Waltham, MA, USA) and washing in phosphate buffer saline (PBS, pH 7.2), followed by a final suspension in PBS. The RBCs in the final suspension were counted with a bench cytometer (ThermoFisher Scientific, Waltham, MA, USA) and provided a density of ~6 × 10^9^ cells/mL. The RBCs were kept on ice but brought to room temperature before initiating the experiments. The RBCs were stained with Laurdan or DPH (both from ThermoFisher Scientific, Waltham, MA, USA) for fluorescence measurements. Laurdan and DPH powders were solubilized at 1 mM final concentration in DMSO (Sigma-Aldrich, St. Louis, MO, USA). For complete solubilization, the mixtures were vigorously vortexed, followed by bath sonication for ten minutes. The stock solutions were kept away from direct light and exposed to additional vortexing and one minute of sonication before each use. For experiments employing fluorescence measurements, 2 mL of RBC stock solution were stained with Laurdan or DPH at 5 µM final concentration, rotated in a test tube for two hours at room temperature, and washed three times with PBS by centrifugation. The final pellet was reconstituted in 2 mL PBS, and aliquots from it were used for cholesterol extraction, exposure to hypo-osmotic shock, and exposure to pore-forming toxins. Multiple 2 mL tubes of stained and non-stained RBC stocks were prepared when larger volumes were needed for experiments. In this case, all the pristine stocks were mixed prior to staining and/or experimentation to avoid non-uniformities in each sample’s cellular density.

To deplete the cholesterol in the RBC membranes, we used methyl-β-cyclodextrin [48] (MBCD, Sigma-Aldrich, St. Louis, MO, USA) solubilized in PBS at 250 mM final concentration. The stock solution was briefly sonicated for 30 s before each use in a bath sonicator. Cholesterol extraction from RBCs was performed by combining in test tubes PBS, 25 µL stock of stained RBCs, and MBCD at final concentration ranging from 0 µM (control) to 1000 µM for a total volume of 3 mL. The tubes were rotated in a bench rotator at room temperature for two hours, and the contents were transferred to plastic cuvettes (1 cm path length) for fluorescence measurements.

### 2.2. Pore-Forming Toxins

Two PFTs, lysenin and streptolysin O (SLO), were used for our experiments. Aliquots from a stock solution of oxygen-stable SLO (a kind gift from Dr. Sarah Hobdey, VA, Boise, ID, USA) were serially diluted in PBS to achieve the desired concentrations such that no more than a few µL were added to the RBC solutions. The final concentrations of lysenin and SLO in the samples are indicated in the Results and Discussion. Aliquots from a recombinant lysenin stock solution [57] were serially diluted similarly to SLO. Both toxins were added to test tubes containing 3 mL of PBS and 25 µL stock of stained RBCs. PFT-exposed samples and negative controls without PFTs were rotated for one hour at room temperature in a bench rotator, transferred to cuvettes, and subjected to fluorescence measurements.

### 2.3. Hypo-Osmotic Shock Exposure

A total of 25 µL of stained stock RBCs were added to test tubes containing 3 mL mixtures of PBS and water to achieve the desired hypo-osmotic pressures. The percentages of added water varied from 0% (control) to 80%. After the tubes were rotated for 20 min in a bench rotator at room temperature, the solutions were transferred to cuvettes for fluorescence measurements.

### 2.4. Fluorescence Measurements

The fluorescence was measured with a Fluoromax 4 Spectrofluorometer (Horiba Scientific, Piscataway, NJ, USA). To ensure maximum sensitivity and minimization of experimental errors, we implemented measurement protocols consisting of single-point excitation and one- or two-point emission. For Laurdan, the protocol was defined as follows: 410 nm excitation, 5 nm excitation bandpass, emission one at 440 nm, 10 nm emission bandpass, emission two at 490 nm, 10 nm emission bandpass, and a target measurement error of 1%. Although Laurdan is customarily excited at 350 nm, excitation at longer wavelengths improved the signal/noise ratio, which is theorized to be a consequence of photo-selection [25,31,34]. All fluorescence intensity measurements were carried out in triplicate and at room temperature. The generalized polarization function (GP) was estimated from the ratiometric formula [4,30,33,47,58]:

(1) $$\mathrm{GP}=\frac{I_{440}-I_{490}}{I_{440}+I_{490}}\;$$

The GP values were computed directly in the instrument’s software. Average values and standard deviations were calculated and plotted using Origin 8.5.2 software package (OriginLab, Northampton, MA, USA). Anisotropy was measured with the same instrument equipped with polarizers to determine the fluorescence intensity for the parallel ($I_{∥}$) and perpendicular (I_⟂_) orientations. The anisotropy parameter “p” was measured with the single-point protocol (360 nm excitation, 5 nm bandpass, 450 nm emission, 10 nm bandpass, and 1% error) from the following relation [14,38,59]:

(2) $$p=\frac{I_{∥}-I_{⟂}}{I_{∥}+2I_{⟂}}\;$$

The sigmoidal plots have been fitted in Origin 8.5.2 with the four-parameter Boltzmann function [26,28]:

(3) $$y=\frac{\max-\;\min}{1+e^{\left(x-x_{0}\right)/\mathrm{dx}}}+\min$$

where max and min are the y coordinates of the plateaus, x_0_ is the x-coordinate of the inflex points, and dx is the slope of the corresponding dependence [28].

## 3. Results and Discussion

### 3.1. Cholesterol Depletion in Red Blood Cell Membranes Modulates Lipid Ordering

Our initial investigations focused on understanding the response to cholesterol reduction in RBC membranes at room temperature by gradual depletion with MBCD. The effects of the cholesterol content adjustments in artificial and natural membranes have been extensively investigated with respect to the changes in the optical response of membrane probes [26,27,28,29,31,33,60,61]. Many of these experiments were interpreted by exploiting the changes elicited over substantial variations in temperature. We utilized these interpretations to relate the status of the membrane to molecular adjustments of the lipids in response to environmental factors excluding temperature as a variable parameter.

**Figure 1:**
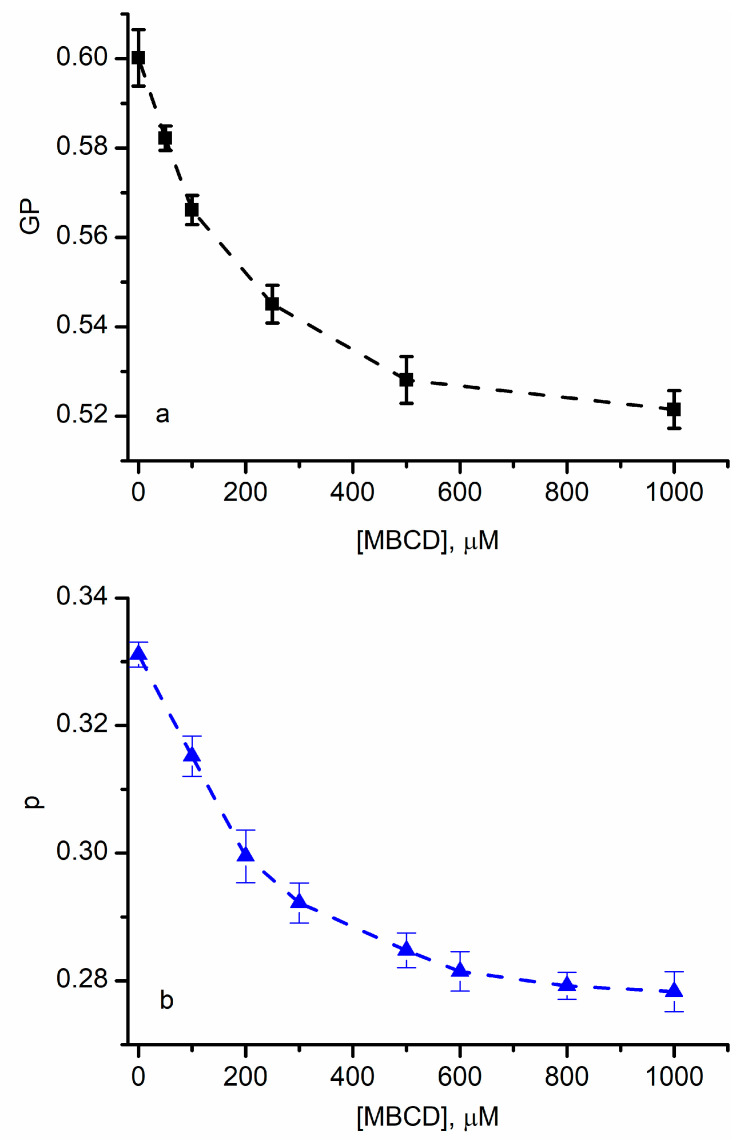
Cholesterol depletion in red blood cell (RBC) membranes adjusts the lipid ordering. The decrease in Laurdan’s generalized polarization function (**a**) and anisotropy of diphenylhexatriene (DPH) (**b**) upon methyl-β-cyclodextrin (MBCD) addition indicates a reduced lipid order in the membranes. The symbols represent average values ± SD (*n* = 3); the interrupted lines are added as visual aids.

Figure 1 shows Laurdan’s generalized polarization and DPH’s anisotropy for RBCs exposed to increasing concentrations of MBCD (up to 1000 µM). The control, 0 µM of MBCD, was characterized by initial values of GP (~0.6) and p (~0.3) typical for artificial and natural membranes at room temperature [26,27,28,29,30,31,32,38,47,48,62]. To interpret the effects of cholesterol, we considered the changes in lipid ordering induced by temperature variations in membranes undergoing phase transitions [26,28,31,32,38,47].

Artificial membrane systems comprising few lipid components with no cholesterol exist in a highly ordered gel phase at low temperature (i.e., under the melting temperature). The increase in temperature leads to melting and the attainment of a liquid-disordered phase, and a clear order–disorder transition is easily observable within narrow temperature ranges [26,27,28,29,30,47,48,63]. The addition of cholesterol prevents the gel phase at temperatures under the melting point, keeping the lipids in a liquid-ordered phase. Complete phase transitions can still be observed in complex artificial and natural membranes with cholesterol, but extreme temperature ranges must be employed [26,28,30,33]. For limited temperature intervals, the mostly linear variation of the optical response prevents ascribing a clear-cut transition temperature while enabling a qualitative analysis in terms of increasing or decreasing ordering [27,28,33].

At room temperature, RBCs are in a liquid-ordered phase, and the extraction of cholesterol is expected to lead to changes in ordering, which translates to changes in the GP and p parameters. We observed that both parameters decreased as the MBCD concentration increased, suggesting that cholesterol depletion led to a reduced order of the lipids in the membrane [4,14,26,28,48].

The determined changes in GP and p were small. However, large variations for similar MBCD concentrations have been obtained only in response to significant changes in temperatures when true phase transitions were recorded [26,48]. If we inspect these prior experiments, we may observe that the reported changes in GP and p near room temperature are similar to our measurements. An additional contributor to these small variations is the fact that 1000 µM of MBCD does not completely remove the cholesterol from the target membranes, resulting in a large fraction of the initial cholesterol concentration still residing in the RBC membranes [48].

Our plots show a monotonic decrease in lipid ordering, but none suggest that a true phase transition occurred at room temperature due to cholesterol depletion since the typical sigmoidal shape of the plots [26,27,28,47,48,63] is not present. Despite the minor changes in GP and p, we can provide an interpretation of our data by accounting for effects of cholesterol at the molecular scale. Although no complete order–disorder transition is observed, and the degree of ordering cannot be determined, both results are consistent with a gradual reduction in the ordering of the lipids (i.e., the lipids become more disordered when the amount of cholesterol decreases). While the DPH’s anisotropy is directly linked to viscosity and fluidity [27,28,36,38,48], the GP parameter is descriptive of dipolar relaxation [5,31,32,34,38,60]. Irrespective of the interpretation of the changes in GP upon cholesterol depletion (i.e., water penetration as opposed to the influence of the internal motions of lipids on the dipolar relaxation in the vicinity of the excited state dipole [25]), the shift in emission and the changes in anisotropy suggest a decrease in ordering.

### 3.2. Hypo-Osmotic Shock Modulates the Optical Responses of Environment-Sensitive Membrane Probes

**Figure 2:**
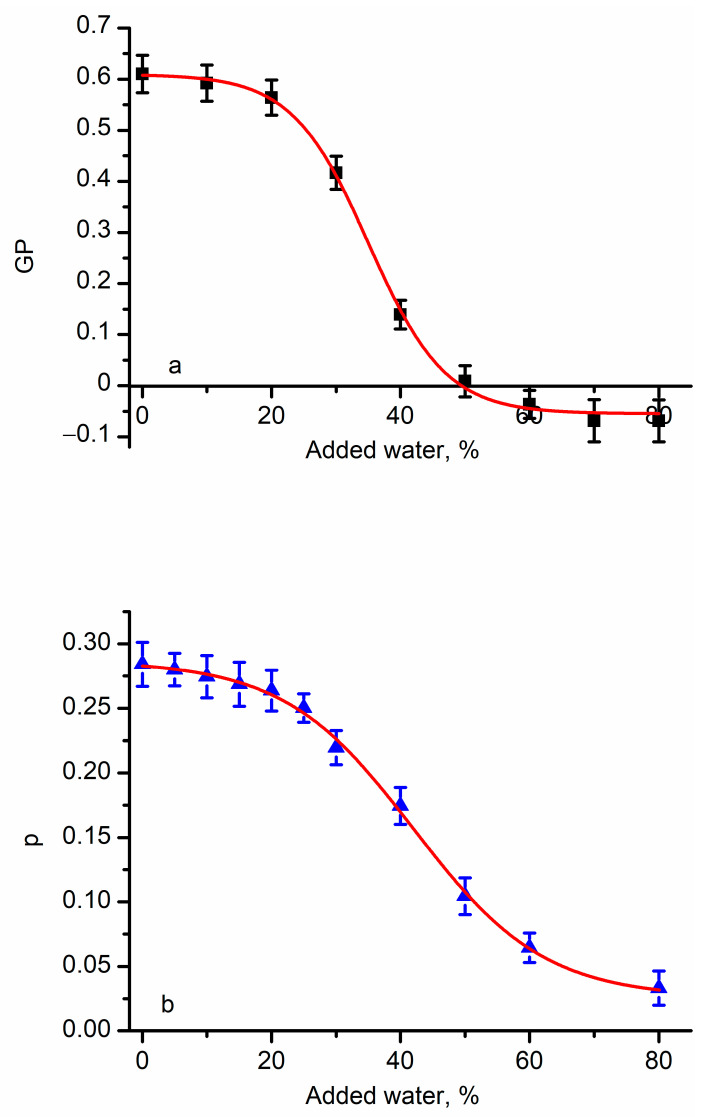
The optical response of membrane probe dyes residing in the membrane of RBCs is modulated by hypo-osmotic pressure and indicates changes in lipid ordering. The generalized polarization of Laurdan (**a**) and DPH’s anisotropy (**b**) decreased in a sigmoidal fashion upon gradual exposure to hypo-osmotic shocks. The symbol represents average values ± SD (*n* = 3); the red traces show the fit with the Boltzmann function, resembling phase transitions.

Our next experiments focus on investigating the physical changes in lipids in cell membranes exposed to hypo-osmotic shock conditions. These investigations filled a large gap in our knowledge [53] in regard to lipid organization since prior explorations employed artificial membrane systems [35,54,55,56,64], hyperosmolarity [53,56], or temperature variations [56]. These experiments built a solid foundation for qualitative analyses of membrane status from the provided interpretations of the changes in the properties of membranes exposed to differential osmotic pressure conditions [55,56,65]. Laurdan and DPH in RBCs’ membranes showed similar patterns in the optical responses upon exposure to hypo-osmotic conditions with respect to changes in generalized polarization and anisotropy (Figure 2).

**Figure 3:**
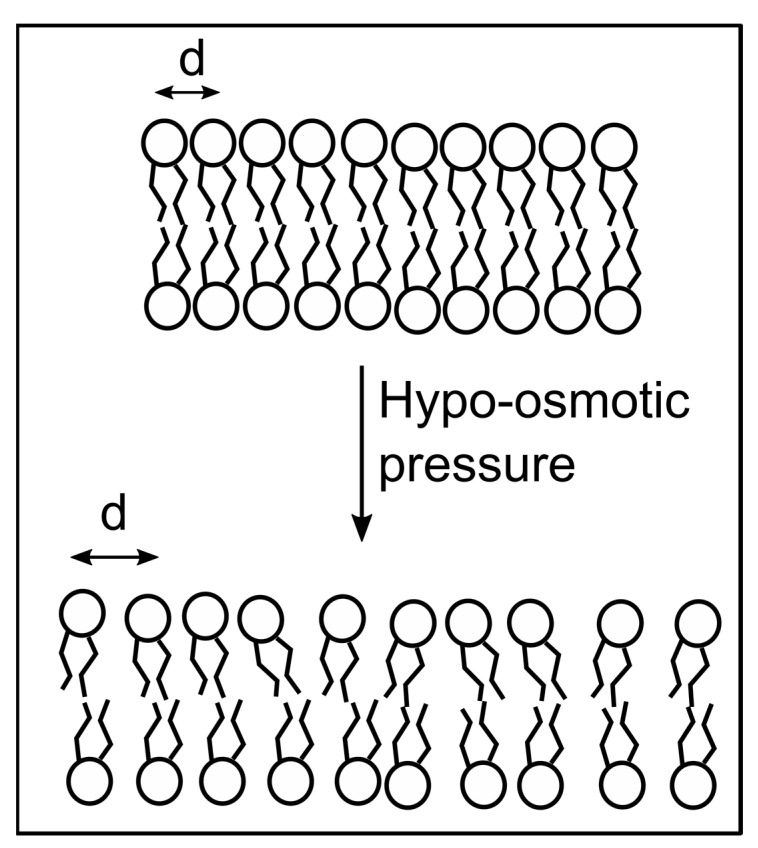
Proposed mechanism for the changes in lipid ordering elicited by membrane exposure to hypo-osmotic pressure. Under hypo-osmotic stress, the intermolecular distance d between neighboring lipid molecules increases. The short-range interactions between the membrane components are overcome by the membrane’s tension induced by stretching, leading to decreased lipid ordering, greater fluidity, and accessible solvation.

The changes in GP and p are much larger than what we measured for cholesterol-depleted RBC membranes. The GP value decreased from ~0.6 to ~−0.1 (theoretical range +1:−1), and DPH’s anisotropy decreased from ~0.28 to ~0.02 (theoretical range +1:0). Both variations are typical for temperature-induced phase transitions [26,28,30,33,48]. For comparison, experiments on giant vesicles and yeast exposed to osmotic pressure conditions exhibited much smaller variations [35,56]. In addition to the large variation of the optical response recorded for RBCs under hypo-osmotic conditions, it was striking that the plots present a pronounced sigmoidal shape resembling true phase transitions similar to the ones determined for membrane systems undergoing significant temperature changes. The resemblance to phase transitions prompted us to apply a Boltzmann fit (Equation (3)) of the experimental data [26,28] to provide insight into the molecular processes accompanying changes in membranes exposed to hypo-osmotic pressure. The fit provided similar halfway values of the osmotic pressure (i.e., ~35% water for Laurdan and ~42% water for DPH). However, the Boltzmann fit is usually employed to provide an assessment of phase transitions and ordering induced by changes in temperature, and the determined halfway values indicate the temperatures at which a transition occurs [26,28]. This strongly suggests that hypo-osmotic pressure induces a major transition from liquid-ordered to liquid-disordered membranes at room temperature. One may argue that the asymptotic plateau observed at extreme hypo-osmotic conditions may be a consequence of complete membrane disintegration. However, membrane resealing after a rupture caused by hypo-osmotic shock is well documented [66,67], and our images (Supplementary Material) suggest the existence of intact membranes after applying hypo-osmotic shocks and PFT treatments. To explain this unexpected behavior in membranes containing cholesterol, we accounted for the potential relationships between lipid order, fluidity, and interactions in the membrane. Basically, all the intermolecular forces between the components of the membrane have a short range [68]. Hypo-osmotic pressure leads to water transport into RBCs, swelling, increased membrane tension, an increased surface area, and an increase in the intermolecular distances [44,52,55,65,68,69,70] (as depicted in Figure 3). Consequently, the intermolecular forces were diminished dramatically because of their strong dependency on distance, the lipid order decreased, and the membrane became more fluid, which opposed what was observed for cells exposed to hyperosmotic pressure [53,56].

The hypothesized changes in intermolecular distances and lipid ordering may explain water penetration, which adjusts the Laurdan’s emission, and the effects on DPH’s rotational diffusion. The rotational diffusion depends on the environment’s viscosity and temperature; however, our experiments were performed at a constant temperature. Therefore, the increased intermolecular distances and less rigid membrane may allow faster adjustments of the DPH’s orientation, hence modulating the anisotropy as observed in our experiments.

### 3.3. Pore-Forming Toxins (PFTs) Adjust the Lipid Order in Target Membranes

**Figure 4:**
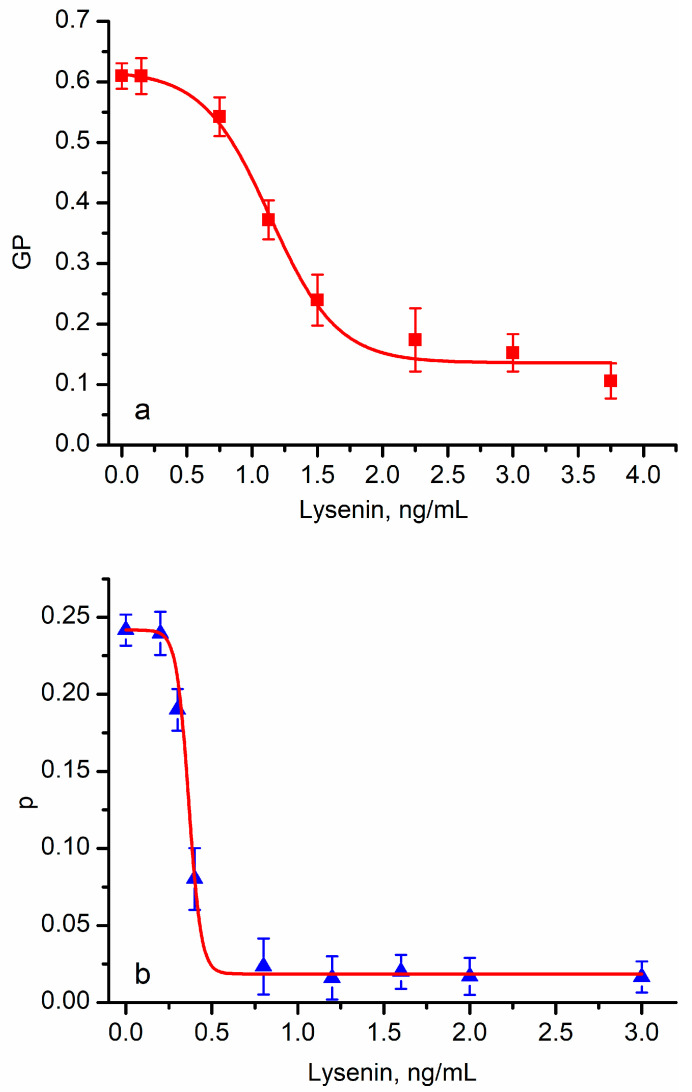
Lysenin insertion into RBC membranes decreases lipid ordering. The Laurdan’s generalized polarization function (**a**) decreased upon lysenin addition in a concentration dependent manner, suggesting that channel insertion led to a reduced ordering of the lipids. A marked decrease was also encountered for DPH’s anisotropy upon lysenin’s insertion (**b**), supporting the hypothesis of changes in lipid ordering. Each symbol represents average experimental data ± SD (*n* = 3), and the red traces show the fit with the Boltzmann function.

Next, we addressed the changes in lipid order induced by the insertion of two model PFTs, lysenin and SLO, into RBC membranes. The lysenin addition led to a gradual decrease in the GP values (Figure 4a), which was interpreted based on the Laurdan’s ability to indicate lipid order, fluidity, and solvent exposure. The GP decreased significantly (from ~0.6 at 0 ng/mL of lysenin to ~0.1 at 3 ng/mL of lysenin) as lysenin’s concentration increased, indicating that the addition of a toxin led to a faster dipolar relaxation. Similarly, the DPH’s anisotropy decreased considerably (Figure 4b) from ~0.24 at 0 ng/mL of lysenin to ~0.02 at 0.6 ng/mL of lysenin. Both variations were larger than the ones induced by cholesterol depletion (Figure 1) and approached the changes measured for hypo-osmotic conditions (Figure 2), suggesting the occurrence of major changes in the RBCs’ membrane status.

**Figure 5:**
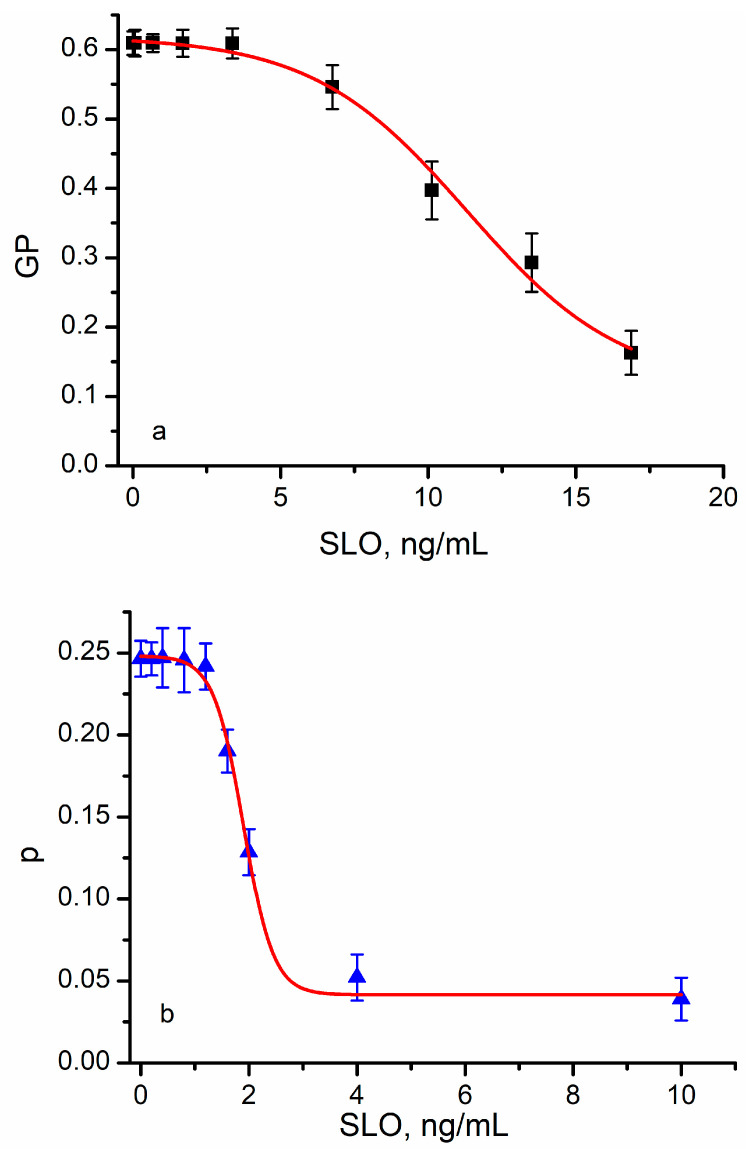
Streptolysin O (SLO) insertion into RBC membranes influences lipid ordering. Upon SLO insertion, Laurdan’s GP function decreased in a concentration-dependent manner (**a**), suggesting that channel insertion decreased ordering of the lipids. A marked decrease was also determined upon SLO’s insertion from DPH’s anisotropy (**b**), suggesting changes in lipid ordering. Each symbol represents average experimental data ± SD (*n* = 3), and the red traces show the fit with the Boltzmann function.

In addition to the larger variations in the optical responses, the exposure to lysenin led to sigmoidal-shaped curves, which were characteristic of phase transitions [26,28]. However, the visual analysis of the plots and the Boltzmann fit indicated a quantitative difference; the halfway concentration of lysenin (the inflex point) was different for the two dyes (i.e., ~1.1 ng/mL for Laurdan and ~0.4 ng/mL for DPH). DPH’s anisotropy is a direct consequence of the rotational diffusion and, hence, a measure of fluidity. However, Laurdan’s fluorescence stems from its dipolar relaxation, which may have multiple origins. For example, a local change in the lipid ordering by the inserted protein may have a more pronounced influence on the rotational diffusion than water penetration or dipolar relaxation. To further examine the influence presented by toxin insertion, we repeated the experiments by replacing lysenin with another potent pore-forming toxin, streptolysin O (SLO). Strong qualitative similarities between lysenin (Figure 4) and SLO (Figure 5) have been observed in regard to optical response. SLO addition also led to a decrease in generalized polarization and anisotropy in a concentration-dependent manner similar to our lysenin observations. In addition, the absolute changes in GP and anisotropy resembled the values determined for lysenin.

The Laurdan’s response to SLO addition was smaller than the response prompted by lysenin, and a similar decrease in GP was determined for much larger SLO concentrations (i.e., ~16 ng/mL of SLO as opposed to ~3 ng/mL of lysenin). This large difference may not have been a consequence of SLO’s lower biological activity since DPH was more sensitive and showed an optical response similar to lysenin for the same concentration range (the minimum plateau was reached at ~4 ng SLO/mL). Although the sigmoidal shape of the anisotropy was obvious for DPH, Laurdan did not exhibit a strong sigmoidal curve. The inflection points determined from the fit were 11.32 ng/mL for Laurdan and 1.9 ng/mL for DPH. Both dyes are indicative of potential phase transitions occurring due to the protein–membrane interactions, which we exploited to provide a mechanistic view of the changes in membrane status upon exposure to PFTs.

The notable changes in the optical response and the sigmoidal behavior suggest that the RBC membranes exposed to PFTs undergo major changes at the molecular level. The functional pore is a result of many processes of primary interactions with channel-forming membrane components, such as cholesterol or sphingomyelin, and oligomerization [71,72,73,74,75,76,77]. Both toxins act quickly on target membranes; therefore, the time frame of the experiment allowed for the full equilibration of each sample. No significant changes have been observed for either toxin at low concentrations. A low concentration of the toxins may not preclude interactions with the membranes, but it is uncertain if minimal changes in the optical response are a consequence of the poor sensitivity of the measuring method or the absence of insertion and pore formation. Both toxins elicited large variations in the optical response at high concentrations, which is indicative of notable changes in the membrane’s environment. However, asymptotic behavior, irrespective of the optical probe and approaching a minimal value, was observed for lysenin. SLO presented this behavior only in the presence of DPH. We may not exclude the existence of a similar plateau for the SLO/Laurdan system at SLO concentrations greater than those used in our study. These quantitative and qualitative differences may be explained by accounting for different biological activities (i.e., affinities for target membranes) and the mechanisms of action for the two toxins (i.e., a different mechanism of oligomerization, pre-pore formation, binding to lipid components, and insertion). Nonetheless, major changes in the optical responses were triggered for both toxins at certain concentrations.

**Figure 6:**
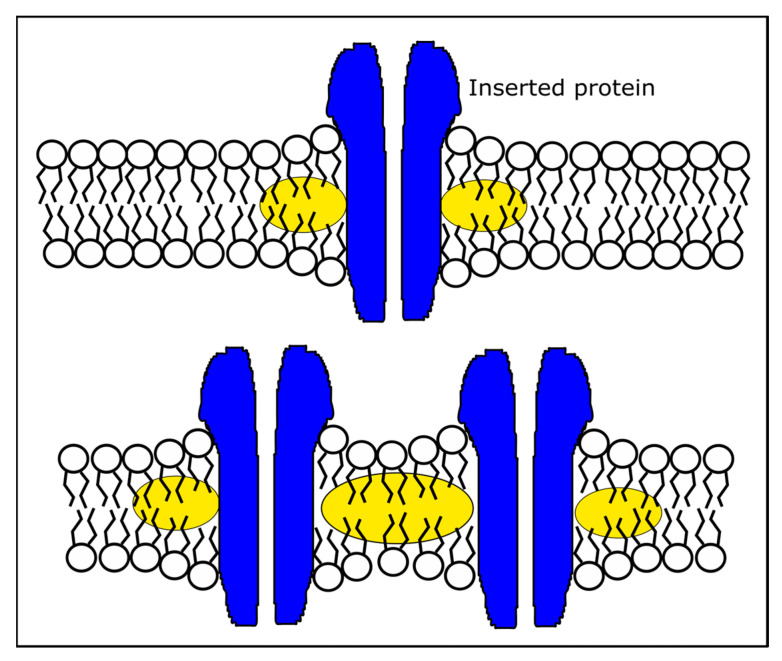
Proposed hydrophobic mismatch mechanism for changes in lipid ordering induced by insertion of pore-forming toxins (PFTs) into target membranes. At low PFT concentrations (**top**), the hydrophobic mismatch locally affects the lipid ordering within a small region surrounding the inserted protein (highlighted ellipses); consequently, the changes in lipid ordering are spatially limited. At high PFT concentration (**bottom**), the inter-channel region is also affected, the disordered volume increases significantly and leads to larger adjustments in dipolar relaxation and rotational diffusion of environmentally sensitive probes.

An explanation of the sudden changes induced by the toxin’s insertion into target RBC membranes is the consideration of boundary conditions at the lipid–protein interface. Inclusions into membranes may manifest by midplane bending, torsional effects, or hydrophobic mismatches [70,78,79,80,81,82,83]. Among these, the structural data of SLO and lysenin [74,84,85,86] suggest that a hydrophobic mismatch leads to the observed changes in generalized polarization and anisotropy. At a low protein concentration, the center-to-center distance between inserted proteins is large. Consequently, substantial membrane deformation may be limited to the immediate region surrounding the inserted protein [70,79,80,81] (Figure 6). This insertion-induced membrane curvature manifests at a short-length scale, and the lipid order is disturbed only within the small volume surrounding the inserted channel. In this small volume, solvent penetration and dipolar relaxation are limited. As opposed to DPH, which undergoes enhanced rotational diffusion in the vicinity of the protein, Laurdan’s relaxation may not benefit from hydration, explaining the weaker response. To explain the large differences in the Laurdan’s response to lysenin and SLO, we may consider the different geometries of the inserted proteins and the different interactions with membrane’s components. Lysenin’s oligomerization and subsequent pore formation requires a primary interaction with sphingomyelin [72,73,85], while SLO binds to the cholesterol molecules inside the membrane [76,77,87]. Consequently, SLO may sequester cholesterol molecules around the inserted channel, preventing major lipid disordering, a decrease in fluidity, and water penetration, thus weakening the optical response to SLO insertion. This effect should also influence the rotational diffusion of DPH, which is suggested by the slightly reduced response elicited upon exposure to SLO in comparison to lysenin.

The weaker response presented by Laurdan in the presence of SLO may also be explained by a higher local concentration of cholesterol upon sequestration. The increased rigidity of the membrane may reduce the lipid motion and prevent water penetration, a major mechanism of Laurdan’s dipolar relaxation. However, higher densities of reconstituted proteins may further contribute to substantial changes in the membrane’s geometry. At reduced center-to-center distances, the curved surfaces may coalesce [70,80,81], leading to larger deformations of the membrane, a global increase in the membrane thickness, greater lipid disorder, and a larger portion of the membrane being prone to solvent penetration. The SLO data are in agreement with tube extrusion experiments that show a loss of the elasticity of SLO’s permeabilized membranes [88]. To explain the differences between SLO and lysenin data, one may consider the lysenin’s preference for insertion into lipid rafts [72,89], which may lead to much higher local densities of proteins in the target membranes.

An additional observation can be made regarding the initial values of GP and p parameters. While these values are consistent with prior reports, the initial anisotropy parameter underwent large variations, while the GP value remained practically the same. One may assume that this experimental variability may originate in the “aging” of RBCs preserved in vitro and our inability to perform all the experiments in a short period of time. Because only anisotropy exhibited large variations, it suggests that the optical responses from the two dyes are not highly correlated. This assumption is reasonable since the two dyes, although sparingly used to assess lipid ordering, respond to different membrane conditions (i.e., dipolar relaxation as opposed to fluidity).

In conclusion, our experiments show that Laurdan and DPH may be used to assess changes in the membrane status induced by hypo-osmotic pressure or the insertion of transmembrane proteins. For both environmental conditions, the variations in the optical response resemble transitions between liquid-ordered and liquid-disordered phases. While such transitions are typically encountered in response to variations in temperature, our results show that they may occur at constant temperature under the influence of mechanical or biological stimulation. We related the presumed phase transitions to the disorganization of the lipids and short-range interactions in the membrane modulated by mechanical stress elicited by hypoosmotic shocks or hydrophobic mismatches in the target membranes. Nonetheless, further work is needed to concretely establish that the observed changes are consequences of true phase transitions in the membranes. Therefore, this work may lead to a better understanding of how cells respond to physical, chemical, or biological environmental stimulations.

## Data Availability

The data presented in this study are available on request from the corresponding author.

## Supplementary Materials

The following supporting information can be downloaded at: https://www.mdpi.com/article/10.3390/membranes13070620/s1, Figure S1: Intact membranes are indicated by fluorescence microscopy.
