# Supplementary material for: Hypo-Osmotic Stress and Pore-Forming Toxins Adjust the Lipid Order in Sheep Red Blood Cell Membranes
Source: Membranes (Basel). 2023 Jun 25;13(7):620. doi: 10.3390/membranes13070620 (PMC10385129; doi:10.3390/membranes13070620)
Supplement: Supplementary file 1 [file membranes-13-00620-s001.zip › membranes-2357099-supplementary.pdf]

Supplementary Material

Hypo-Osmotic Stress and Pore-Forming Toxins Adjust the Lipid Order in Sheep Red Blood Cell Membranes

Rose Whiting [1,2], Sevio Stanton [1], Maryna Kucheriava [1], Aviana R. Smith [1], Matt Pitts [1,2], Daniel Robertson [1], Jacob Kammer [1,3], Zhiyu Li [1] and Daniel Fologea [1,2,*]

[1] Department of Physics, Boise State University, Boise, ID 83725, USA
[2] Biomolecular Sciences Graduate Program, Boise State University, Boise, ID 83725, USA
[3] Department of Family Medicine, Idaho College of Osteopathic Medicine, Meridian, ID 83642, USA
* Correspondence: danielfologea@boisestate.edu

We employed microscopy imaging to verify the presence of intact membranes after various treatments and demonstrated that the observed decreases in general polarization and anisotropy parameters do not originate in membrane disintegration. The visibility of sheep Red Blood Cell (RBC) membranes lacking hemoglobin due to osmotic shock or membrane perforation was improved by staining the membranes with the lipohilic dye 5-hexadecanoylaminofluorescein (1.2 µM final concentration). The images were taken with an Olympus IX-71 microscope equipped with filter sets suitable for fluorescein detection. Control samples consisted of stained RBC suspensions with no treatment. The experimental treatments were hypo-osmotic shock, Methyl-β-Cyclodextrin (MBCD), and the Pore-Forming Toxins (PFTs) lysenin and Streptolysin O. For all treatments, the maximum stresses described in the article (i.e., hypoosmotic shock, MBCD, and PFT concentrations) have been employed. The RBCs were prepared using the same concentrations and protocols described in the materials and methods.

As Figure S1 shows, intact membranes were present in all samples irrespective of the applied treatment. Additionally, no substantial changes in size were observed.

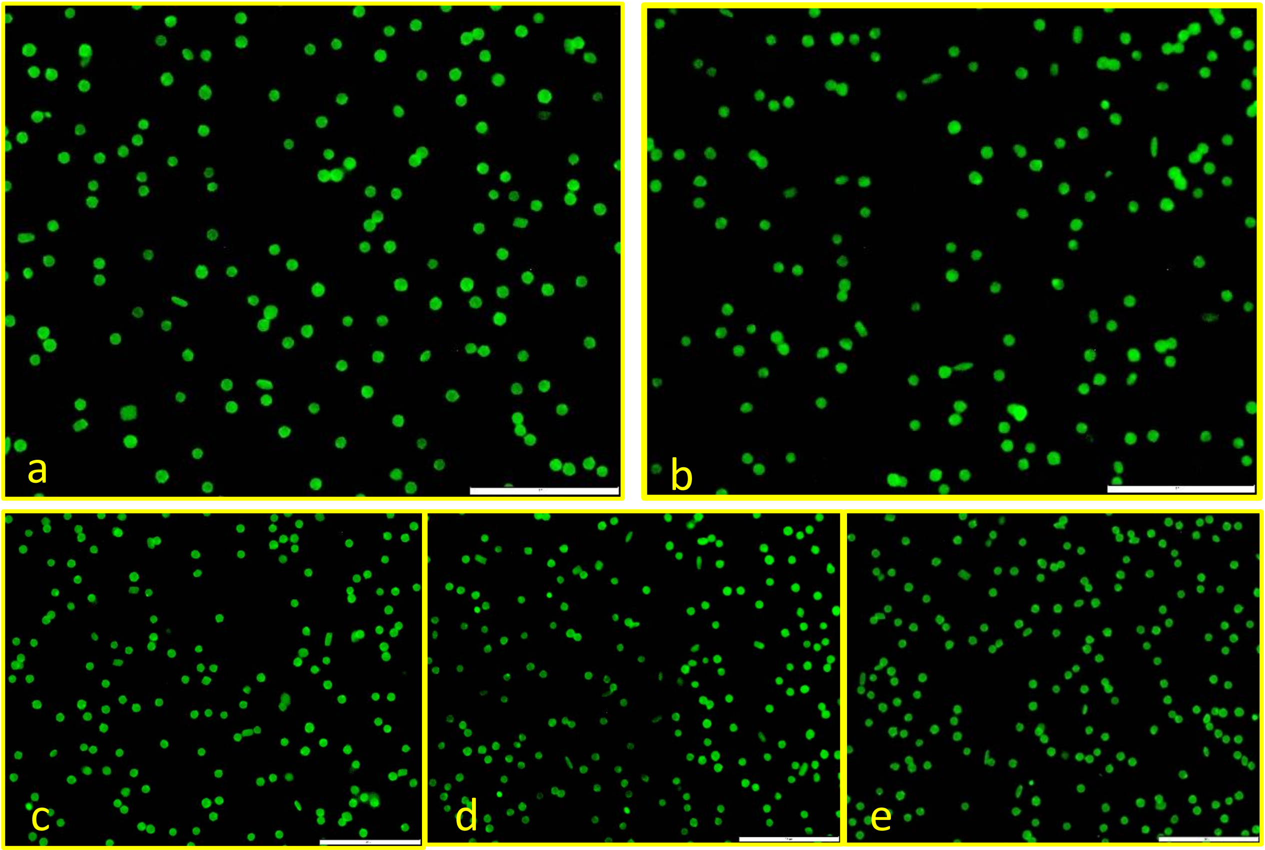

Figure S1. Intact membranes are indicated by fluorescence microscopy of control (a), MBCD (b), hypo-osmotic shock (c), lysenin (d), and Streptolysin O (e) exposed Red Blood Cell samples. The scale bar is 50 µm.
